# Post‐Traumatic Cochlear and Vestibular Pneumolabyrinth

**DOI:** 10.1002/ccr3.71731

**Published:** 2026-01-02

**Authors:** Santiago Almanzo, Catalina Bancalari‐Díaz, Miguel Saro‐Buendía, Vanesa Pérez‐Guillén, Abel Guzmán‐Calvete, Miguel Armengot‐Carceller, Carlos De Paula‐Vernetta

**Affiliations:** ^1^ Department of Otolaryngology Hospital Universitari i Politècnic La Fe Valencia Spain; ^2^ Department of Surgery, Faculty of Medicine and Dentistry University of Valencia Valencia Spain

**Keywords:** cochlear trauma, pneumolabyrinth, sensorineural hearing loss, temporal bone fracture, vestibular dysfunction

## Abstract

Cochlear pneumolabyrinth is a strong radiologic marker of irreversible hearing loss after otic capsule trauma. While vestibular symptoms may resolve spontaneously, cochlear air reliably predicts poor auditory recovery. Early computed tomography confirmation guides counseling and timely evaluation for hearing rehabilitation or cochlear implantation, even when surgical exploration is not indicated.

## 1 Clinical Question

What is the prognosis of pneumolabyrinth affecting both cochlea and semicircular canals following head trauma?

## 2 Case Description

**FIGURE 1:**
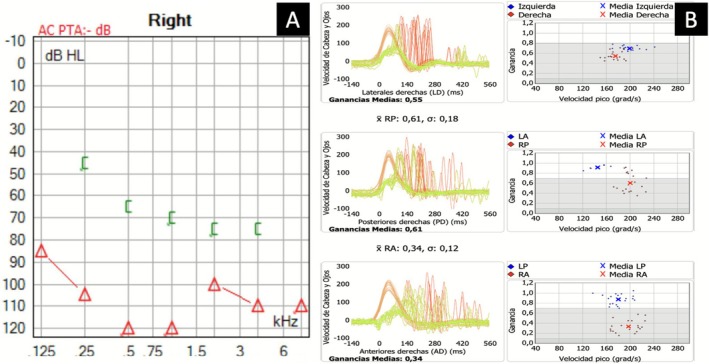
(A) Pure‐tone audiogram: Profound right sensorineural hearing loss. (B) vHIT: Reduced VOR gain in right semicircular canals (lateral: 0.55, posterior: 0.61, anterior: 0.34).

A 21‐year‐old male presented after blunt occipital trauma during an assault. He reported sudden‐onset vertigo, unsteadiness, and right‐sided hearing loss. Otoscopic examination revealed a right hemotympanum. Pure‐tone audiometry showed profound sensorineural hearing loss (Figure 1A). Video Head Impulse Test (vHIT) demonstrated decreased vestibulo‐ocular reflex (VOR) gains in all right semicircular canals (Figure 1B).

**FIGURE 2:**
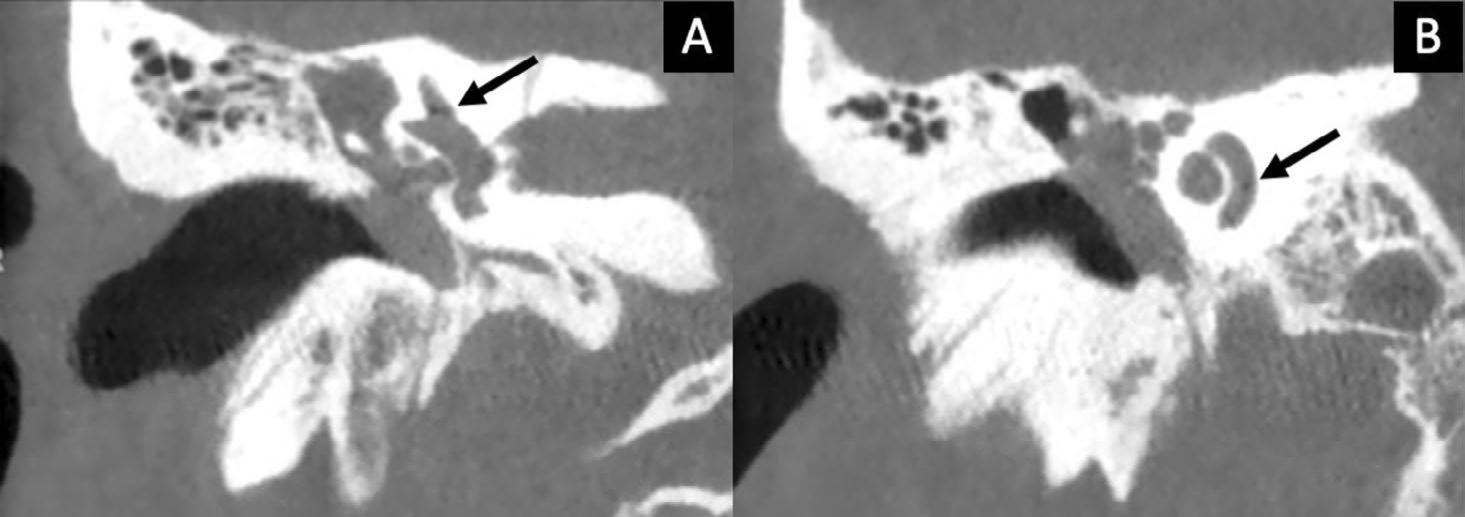
(A) CT: Transverse petrous bone fracture with air in the superior semicircular canal. (B) Air bubbles in the cochlear basal turn.

High‐resolution computed tomography (CT) of the temporal bone revealed a transverse fracture involving the right otic capsule. Air bubbles were detected in both the superior semicircular canal (Figure 2A) and basal turn of the cochlea (Figure 2B), consistent with pneumolabyrinth. Facial nerve function was preserved. Magnetic resonance imaging (MRI) was not performed because CT findings were sufficient for management, although MRI may help identify intralabyrinthine hemorrhage or early fibrosis in selected cases.

The patient was admitted and received intravenous methylprednisolone (1 mg/kg/day for 5 days) followed by oral tapering over 3 weeks, alongside prophylactic antibiotics. Surgery was not indicated because neither the fracture configuration nor the clinical course suggested an active perilymphatic fistula. Exploratory tympanotomy is generally reserved for patients with persistent or progressive symptoms. Vestibular symptoms resolved, but hearing remained profoundly impaired. A cochlear implant was offered but declined. Vestibular recovery was confirmed clinically through complete resolution of vertigo and imbalance; no follow‐up objective vestibular tests were performed.

## 3 Answer and Discussion

Pneumolabyrinth is defined as the presence of air in the inner ear, typically due to a perilymphatic fistula from temporal bone trauma. It may also occur from non‐traumatic causes, including iatrogenic and spontaneous perilymphatic fistulas. The location of air bubbles strongly correlates with prognosis: air confined to the vestibular apparatus may be reversible, while air within the cochlea—especially the basal turn—usually indicates permanent sensorineural hearing loss [1, 2, 3]. Prognosis is also influenced by air volume and associated injuries such as hemorrhage or early ossification. In our case, cochlear involvement predicted poor auditory recovery despite early medical management. Systemic corticosteroids were administered according to common practice in acute temporal bone trauma to reduce inflammatory edema, although supporting evidence remains limited. Prompt diagnosis via CT and a tailored approach are critical for hearing preservation and symptom control. Cochlear implantation remains feasible after otic capsule trauma but may be complicated by post‐traumatic fibrosis or ossification, supporting early referral when hearing recovery is unlikely. This case is notable for the coexistence of cochlear and semicircular canal pneumolabyrinth with complete vestibular recovery but irreversible auditory loss, highlighting the prognostic dissociation between cochlear and vestibular air.

## 4 Take‐Home Message

Cochlear pneumolabyrinth is a radiologic marker of poor hearing prognosis following otic capsule trauma, even when vestibular symptoms resolve.

## Author Contributions

**Santiago Almanzo:** conceptualization, investigation, writing – original draft. **Catalina Bancalari‐Díaz:** conceptualization, investigation, writing – original draft. **Miguel Saro‐Buendía:** conceptualization, investigation, writing – original draft. **Vanesa Pérez‐Guillén:** formal analysis, methodology, validation. **Abel Guzmán‐Calvete:** formal analysis, methodology, validation. **Miguel Armengot‐Carceller:** supervision, writing – review and editing. **Carlos De Paula‐Vernetta:** formal analysis, methodology, supervision, validation, writing – review and editing.

## Funding

The authors have nothing to report.

## Ethics Statement

Ethical approval was waived by the local Ethics Committee of Hospital Universitario y Politécnico La Fe in view of the retrospective nature of the study and all the procedures being performed were part of the routine care.

## Consent

Written informed consent was obtained from the patient for publication of this case report and associated clinical images. The patient understood the purpose of the publication and has approved the inclusion of anonymized clinical photographs and data for academic dissemination.

## Conflicts of Interest

The authors declare no conflicts of interest.

## Data Availability

Data available on request from the authors.
